# The State of Lunacy in England

**Published:** 1863-10

**Authors:** 


### Art. VIII.—THE STATE OF LUNACY IN ENGLAND.

The Seventeenth Report of the Commissioners of Lunacy records the operations of the Lunacy Board, and the state of the population of asylums and workhouses, during the year 1862. The assent of the Board in that period to plans for several new county asylums and the extension of many county and borough asylums, shows that considerable activity was manifested in extending public provision for the insane. New asylums are about to be erected for the counties of Surrey, Stafford, and the united counties of Carmarthen, Pembroke, and Haverfordwest. Surrey will provide accommodation for 651 patients, Stafford for 200, and the united counties for 212. In an appendix to the Report, the entries of the Visiting Commissioners on the condition of those county and borough asylums which it is thought most desirable to notice, chiefly in reference to certain improvements of detail or arrangement of which the asylums are susceptible or have undergone, are published in detail.

The Criminal Asylum at Broadmoor is in an advanced state of preparation. Dr. Meyer, the late superintendent of the Surrey County Asylum, who has been appointed the chief medical officer (and with whom will be associated two assistant medical officers), has taken up his residence at the asylum, and is now actively engaged in organizing the establishment. The near approach of the completion of the asylum has rendered necessary, for the guidance of the Secretary of State in their disposal, an examination of the criminal lunatics in public and private asylums, except those in Bethlehem Hospital and Fisherton House, who are already under his direct control. As the result of this examination, the Report states that "the cases in which removal to the Criminal Asylum appeared necessary or desirable bore a small proportion to the aggregate number." The Commissioners have advised the retention in the several asylums of the large majority of the patients classed as criminal, influenced mainly by the consideration of the comparatively trivial nature of their offences (not affecting life or person), their long residence in the asylum, their harmless and industrious character, and the vicinity of their relatives and friends. "In the course of these special inquiries," say the Commissioners, "our attention having been drawn to the subject generally, we have been forcibly impressed with the impropriety and absurdity of treating a large number of patients confined under Secretary of State's warrants as of the criminal class, or otherwise than as ordinary lunatics, from which they cannot, upon any sound principle, be distinguished. We think it most desirable that the Secretary of State should be empowered (which he is not at present) to transfer such patients from the criminal to the ordinary pauper class. For this and other objects, the revision and consolidation of the Criminal Lunatics Acts appear to us essential. We think it unnecessary to repeat the views we have long held, and frequently expressed, in reference to the law applicable to that class of the insane, and which are embodied amongst others in our Eighth Report. The urgent necessity will appear upon attention to the probable working of the present Criminal Lunatics Act on the large and rapidly increasing numbers of criminal patients." (p. 10.)

Within the nineteen years, 1844—63, the total increase of criminal lunatics has been 620—males 475, and females 145. The total number of criminal lunatics on the 1st January, 1863, was 877—males 677, and females 200; and they were distributed in the following manner:—

| | Males. | | Females. | | Total. |
|---|---|---|---|---|---|
| Asylums | 304 | ... | 115 | ... | 419 |
| Hospitals— | | | | | |
| Bethlehem | 112 | ... | 20 | ... | 132 |
| Other | 8 | ... | 0 | ... | 8 |
| Licensed Houses— | | | | | |
| Metropolitan | 19 | ... | 3 | ... | 22 |
| Provincial— | | | | | |
| Fisherton House | 225 | ... | 61 | ... | 286 |
| Other | 9 | ... | 1 | ... | 10 |

This enumeration is limited to patients under Royal or Secretary of State's warrants in asylums, registered hospitals, and licensed houses. The Commissioners are not in a position to state even approximately the numbers of insane and imbecile inmates in Convict Prisons, the Criminal Hospital at Woking, and in gaols, all of whom the Secretary of State is empowered to send to the asylum at Broadmoor, or any other asylum which may be hereafter provided or appropriated, by warrant under the Royal sign-manual, for criminal lunatics.

The *Judicial Statistics* for 1862 state that the whole number of criminal lunatics under detention in the year ending September 29th, was 1017, of whom 154 were discharged or removed by death or otherwise; 863 (673 males and 190 females) remaining at the end of the twelve months.

The original judgments or orders under which the lunatics in custody in 1862 were detained were as follows:—

| | |
|---|---|
| Found insane | 144 |
| Acquitted insane | 156 |
| Insane, committed by justices | 84 |
| Dangerous lunatics, committed by justices | 3 |
| Convicts becoming insane after trial, and removed by order of Secretary of State | 630 |
| | 1017 |

The periods for which the lunatics have been under detention, and the total number of persons under each period of detention, are thus stated:—

| | |
|---|---|
| One year and under | 222 |
| Two years and above one | 142 |
| Three years and above two | 157 |
| Five years and above three | 140 |
| Ten years and above five | 178 |
| Fifteen years and above ten | 96 |
| Twenty years and above fifteen | 49 |
| Above twenty years | 33 |
| | 1017 |

The offences with which the lunatics were charged, and the number confined for each offence, are thus enumerated:—

| | M. | F. | Total |
|---|---|---|---|
| Murder . . . | 92 | 47 | 139 |
| Attempts to murder, maim, stabbing, &c. | 87 | 13 | 100 |
| Concealing birth, infanticide . . . | ... | 3 | 3 |
| Manslaughter . . | 6 | 4 | 10 |
| Rape . . . . | 8 | ... | 8 |
| Assault with intent to ravish . . . | 12 | ... | 12 |
| Unnatural offences . | 11 | ... | 11 |
| Treasonable and seditious offences . . | 5 | ... | 5 |
| Assaults . . . | 40 | 6 | 46 |
| Indecent exposure of the person . . . | 7 | ... | 7 |
| Burglary and housebreaking . . . | 56 | 4 | 60 |
| Robbery on the highway, &c. . . . | 10 | ... | 10 |
| Sheep-stealing . . | 8 | ... | 8 |
| Horse-stealing . . | 9 | ... | 9 |
| Larceny and petty thefts | 167 | 80 | 247 |
| Frauds and embezzlements . . . | 10 | 1 | 11 |
| Receiving stolen goods | 4 | 1 | 5 |
| Arson and malicious burning . . . | 44 | 4 | 48 |
| Wilful damage and other malicious offences . . . | 10 | 11 | 21 |
| Forgery . . . | 5 | ... | 5 |
| Uttering counterfeit coin . . . | 7 | 3 | 10 |
| Riot and breach of the peace . . . | 27 | 8 | 35 |
| Under the vagrant laws | 39 | 14 | 53 |
| Dangerous persons at large . . . | 3 | ... | 3 |
| Insane, wandering abroad without control | ... | 1 | 1 |
| Deserters from army and navy . . | 8 | ... | 8 |
| In want of sureties . | 50 | 10 | 60 |
| Other offences . . | 61 | 21 | 82 |
| Total | 786 | 231 | 1017 |

The Commissioners dwell upon the advantages of lunatic hospitals to the poor and middle classes who can ill-afford to defray the expense of maintaining insane relatives in a private asylum. They describe them as benevolent institutions, "free from the objection incident to pecuniary interest in the patient;" yet they refer, without animadversion, to a recent attempt to found such hospitals by forming joint-stock associations on the principle of limited liability! Again, looking upon hospitals in this light, they say, "it would appear desirable to render admission into them less difficult, if not in every case, yet at least in those where the patients suffer from sudden and uncontrollable impulses to commit violence or suicide, and *who desire to place themselves under care and treatment.*"

Further, the Commissioners say:—

"But besides persons of unsound mind, for whose medical care and treatment hospitals are primarily intended, it has occurred to us that it would be very desirable if arrangements were made for the reception therein of persons, of whom we have reason to know there are many, not insane, who, being conscious of a want of power of self-control or of the addiction to intemperate habits, or fearing an attack or a recurrence of mental malady, and being in all respects free agents, are desirous of residing as voluntary boarders in an institution for the insane. Upon the question whether, and, if so, in what way, such arrangements can be legally and properly carried out, we have thought it well to fortify ourselves by the opinion of a counsel of eminence, and we accordingly submitted a case to Mr. Welsby, a copy of which, and of his opinion, will be found in the Appendix. It will be seen that he entirely concurs with us in our construction of the enactments upon the subject; that there is nothing in the statutes to prevent the admission of the persons referred to as voluntary boarders into registered hospitals; and that there would be no difficulty in enforcing legally the stipulations and conditions of any agreement, by bond or otherwise, for their residence therein." (p. 13.)

The importance of these suggestions cannot well be exaggerated. The necessity of some relaxation of the law, to meet the classes of cases referred to, has been again and again urged for many years in the pages of this journal, and in the writings of every authority on the subject of lunacy in the kingdom. We hail, then, with great gratification this indication that the Commissioners are beginning at length rightly to appreciate the great moment of making some provision for an order of patients who, the victims of morbid impulse, or consciously verging on insanity, have hitherto been deprived by a needless and barbaric stringency of the law, of the most effectual means of relief—of the right of proper and efficient treatment—to the danger of the public welfare, the incalculable and unnecessary injury of individual and family interests, and the augmentation of insanity. It is an absurdity to presume that the suggestions of the Commissioners could be restricted to any one class of institutions for the insane.

The Commissioners justly lament the deficiency in England of the kind of accommodation provided for lunatics by the existing lunatic hospitals, and the little sympathy manifested by the public in providing additional hospitals. In order to aid those who may be desirous to extend this kind of provision for the insane, they have endeavoured to estimate the probable outlay necessary in land and buildings. The Manchester hospital, standing on 52 acres of land, and accommodating 100 patients, cost, it would appear, 30,208*l.* The Coton Hill institution, near Stafford, accommodates 140 patients, occupies a site of 30 acres, and cost 39,926*l.* 11*s.* 11*d.* The outlay in the seven chartered asylums in Scotland (which are analogous to the English hospitals) amounted, in the year 1855, to 352,632*l.* For this sum, provision was made in buildings, furniture, and land (216 acres) for 2163 patients, 642 private and 1511 pauper patients.

The Commissioners direct attention to the want of a hospital for insane soldiers, such as is provided for the insane of the navy. They express hopes of a fair prospect for the removal of

Bethlehem Hospital into the country; but the recent decision of the Governors of St. Thomas's Hospital to accept a site for their new hospital on the ground to be recovered in part from the Thames, opposite the Houses of Parliament, will, it is to be feared, postpone indefinitely the chances of the Commissioners' hopes being fulfilled. But even if this be the case, the strictures of the Commissioners upon the site, not only of Bethlehem, but also of St. Luke's, are not in the least weakened, as also upon the gloomy aspect of the last-named hospital.

The visiting Commissioners report very favourably of the improved management of the Asylum for Idiots at Earlswood. They had previously recommended that the mode of training the patients should be altered, by diminishing the amount of scholastic tuition, and giving greater prominence to physical exercise and manual employment. "Since our colleagues visited, on the 18th June in last year," they say, "there have been 60 admissions, 31 discharges, and 21 deaths. . . . . . . . Examination of the results in the discharged cases gives satisfactory proof of the beneficial working of the institution. But of the 31, only two were sent away wholly unimproved; 17, 10 boys and 7 girls, were improved—the majority of these greatly; and 12, 7 boys and 5 girls, had so much profited by the instruction obtained here as to be able to work for their livelihood, some of them having since got regular employment—the girls in domestic service, and the boys as carpenters, tailors, and mat makers." (p. 17.)

The modifications which have been introduced with such excellent effect into the system of the Earlswood Asylum have also been introduced with advantage into the Eastern Counties Asylum for Idiots and Imbeciles at Colchester.

No material difference is reported in the condition of private asylums, and but three cases of discipline are recorded—one arising out of the death of a patient suffering from acute mania, and who died in consequence of injuries received in a struggle with his attendants; the second connected with the well-known case of *Hall* v. *Semple*; the third a case of detention without order and medical certificates. In the first case, the coroner's jury exonerated the attendants from blame, but the Commissioners express an opinion that there had been a want of judicious care and supervision. In the second case, the Commissioners reprehend in the strongest terms the conduct of the proprietor of the asylum, he having received Mr. Hall under charge on manifestly invalid certificates, as the gentleman who signed the second certificate had failed in complying with the requirements of the statute. In the third case, the offending proprietor was prosecuted in the Court of Common Pleas, and pleading guilty, entered into recognizances to appear and receive judgment whenever called upon to do so.

The Commissioners have recently issued a revised order, of which a copy is given in the Appendix to the Report, directing the form of particulars to be from time to time entered in the Medical "Case-book." They recur to the question of the selection and remuneration of attendants, and propose to renew their inquiries on this subject during their next statutory visitations. They direct attention also to the advantages which patients derive by a temporary absence from asylums, and they think it very desirable that the superintendents of Public Asylums should be invested with discretionary authority in this matter, without requiring, in every case, a special order by two Visitors. The visiting Commissioners observe, of the Gloucester County Asylum, that "the practice of allowing patients to visit their friends for the day, and occasionally for a few days, is reported to be attended with the best results. Upwards of four hundred of such visits are stated to have been made during the past year, and in no case has the written undertaking to bring back the patient within the stipulated time been violated."

Of workhouses, the Commissioners report that little improvement has taken place in the treatment and accommodation of the insane poor lodged in them. Reiterating their opinions on the altogether unsuitable arrangements of workhouses for lunatics, the Commissioners direct attention to certain provisions of the Lunacy Acts Amendment Act of last year, which will materially affect their power and position in reference to the visitation of workhouses. They are hopeful that, by means of the additional powers conferred upon them in this respect, they may be enabled ultimately to remove the "more glaring evils now existing, and, in some degree, ameliorate the condition of the unfortunate class of pauper lunatics who must necessarily remain inmates of the workhouse wards." The 8th section of the Act "enables the visitors of any asylum, and the guardians of any parish or union within the district for which the asylum has been provided, to make arrangements for the reception and care of a limited number of chronic lunatics in the workhouse, subject to the approval of the Commissioners in Lunacy and the President of the Poor Law Board." By the 31st section, two or more Commissioners are empowered, by an order under their hands, "to direct the removal to an asylum of any lunatic therein, who, in their opinion, is not a proper person to be kept in the workhouse;" and the 32nd and 33rd sections further enable them "to visit and examine any pauper lunatic not in a workhouse, and upon the certificate of a medical man, to make a peremptory order for the reception of the patient into an asylum, hospital, or licensed house. The 34th section has reference to the lists of patients in asylums chargeable to unions and parishes; and by the 37th section, the visiting Committee, &c., of unions and parishes are required, at least once in each quarter, to make a report relative to the dietary, accommodation, and treatment of the lunatics in workhouses, which reports are to be laid before the Commissioners on the occasion of their visits."

Applications under the 8th section have already been made to the Commissioners, for their approval of arrangements made, or to be made, in workhouses, for the reception of a stated number of patients, selected by the Superintendents of Asylums as harmless chronic lunatics who may be properly treated therein. In one case only, however, has it been requisite for the Commissioners to give a formal assent; the number of lunatics to be removed in the remaining instances being too limited to require their interference. But the Commissioners have issued the following minute of the conditions on which alone they will grant permission for the removal of patients, where the number is such as to need their intervention.

1. That separate wards be provided for patients of each sex.

2. That the wards contain both day-rooms and dormitories.

3. That the floors of all the rooms be boarded.

4. That at least 500 feet of cubical space be allowed in the dormitories for each patient, exclusive of passages and corridors.

5. That at least 400 feet of cubical space be allowed in the day-rooms for each patient.

6. That the single bed-rooms contain at least 600 feet of cubical space.

7. That the associated dormitories for males contain at least three beds.

8. That each ward be provided with a water-closet, bath-room, and lavatory indoors.

9. That suitable airing-courts be set apart for the exclusive use of the patients.

10. That the wards be properly furnished, and that every male have a separate bed.

11. That at least one paid attendant for each sex be appointed to reside in the wards, and to be exclusively employed in the care of the patients.

12. That arrangements should have been made for visitation by the medical officer, either daily, or so often as the Commissioners shall determine. That he shall keep a record of all admissions, discharges, and deaths, and of cases of seclusion, accidents, and escapes, &c., in a form to be approved of by the Commissioners and the Poor Law Board.

13. That a code of rules and regulations should have been drawn

| | Number of Patients, 1st January, 1862. | | | | | | | Admissions during the Year 1862. | | | Discharges during the Year 1862. | | | | | | Deaths during the Year 1862. | | | | | | | | |
|---|---|---|---|---|---|---|---|---|---|---|---|---|---|---|---|---|---|---|---|---|---|---|---|---|---|
| | Private. | | | Pauper. | | | Total Lunatics. | | | | Total Number. | | | Number Recovered. | | | Total Number. | | | From Suicide. Act committed in Asylum. | | | From Suicide. Act committed before admission. | | |
| | M. | F. | Total. | M. | F. | Total. | — | M. | F. | Total. | M. | F. | Total. | M. | F. | Total. | M. | F. | Total. | M. | F. | Tot. | M | F. | Tot. |
| County and Borough Asylums | 155 | 112 | 267 | 8,756 | 10,631 | 19,387 | 19,654 | 3,099 | 3,046 | 6,145 | 1,503 | 1,671 | 3,174 | 1,096 | 1,318 | 2,414 | 1,137 | 915 | 2,052 | 8 | 4 | 12 | 5 | 3 | 8 |
| Hospitals . | 1,096 | 794 | 1,890 | 127 | 135 | 262 | 2,152 | 455 | 412 | 867 | 290 | 322 | 612 | 154 | 184 | 338 | 106 | 67 | 173 | ... | 2 | 2 | ... | ... | |
| Metropol. Licensed Houses | 781 | 656 | 1,437 | 228 | 467 | 695 | 2,132 | 506 | 499 | 1,005 | 313 | 304 | 617 | 151 | 149 | 300 | 137 | 109 | 246 | 4 | ... | 4 | ... | ... | ... |
| Provincial Licensed Houses . | 923 | 733 | 1,656 | 293 | 312 | 605 | 2,261 | 426 | 361 | 787 | 311 | 324 | 635 | 115 | 141 | 256 | 97 | 58 | 155 | ... | 1 | 1 | 1 | ... | 1 |
| Totals . | 2,955 | 2,295 | 5,250 | 9,404 | 11,545 | 20,949 | 26,199 | 4,486 | 4,318 | 8,804 | 2,417 | 2,621 | 5,038 | 1,516 | 1,792 | 3,308 | 1,477 | 1,149 | 2,626 | 12 | 7 | 19 | 6 | 3 | 9 |

| | Patients Remaining 1st January, 1863. | | | | | | | | | | | | | | | | | | |
|---|---|---|---|---|---|---|---|---|---|---|---|---|---|---|---|---|---|---|---|
| | Private. | | | Pauper. | | | Total Lunatics. | Number deemed Curable. | | | Found Lunatic by Inquisition. | | | Criminals. | | | Chargeable to Counties or Boroughs. | | |
| | M. | F. | Total. | M. | F. | Total | | M. | F. | Total | M. | F. | Total | M. | F. | Total | M. | F. | Total |
| County and Borough Asylums . . . | 149 | 110 | 259 | 9,221 | 11,093 | 20,314 | 20,573 | 902 | 1,209 | 2,111 | 9 | 6 | 15 | 304 | 115 | 419 | 702 | 796 | 1,498 |
| Hospitals . . . . . . . . . . . | 1,127 | 801 | 1,928 | 155 | 151 | 306 | 2,234 | 177 | 189 | 366 | 23 | 23 | 46 | 120 | 20 | 140 | 2 | ... | 2 |
| Metropolitan Licensed Houses . . . | 803 | 645 | 1,448 | 262 | 564 | 826 | 2,274 | 121 | 206 | 327 | 78 | 76 | 154 | 19 | 3 | 22 | 31 | 39 | 70 |
| Provincial Licensed Houses . . . . | 963 | 743 | 1,706 | 271 | 281 | 552 | 2,258 | 170 | 178 | 348 | 79 | 50 | 129 | 234 | 62 | 296 | 33 | 20 | 53 |
| Totals . . . . . . . . . . . | 3,042 | 2,299 | 5,341 | 9,909 | 12,089 | 21,998 | 27,339 | 1,370 | 1,782 | 3,152 | 189 | 155 | 344 | 677 | 200 | 877 | 768 | 855 | 1,623 |

Included in Total Lunatics.

up, setting forth the powers and duties of the medical officer, and for the guidance of the attendants and general management of the wards.

14. That a special and liberal dietary, to be approved by the Commissioners and the Poor Law Board, be fixed.

The condition of single patients, pauper and private, is briefly referred to by the Commissioners, and several cases of difficulty in the right disposal of property of lunatics detailed. The proceedings are also reported, which the Commissioners found it necessary to institute in reference to several cases of deaths from violence, injuries, and defective returns and certificates in county asylums. A brief abstract is given of the Lunacy Acts passed in the last session of Parliament, the provisions of which have been already set forth in these pages,* and the Commissioners terminate their Report with some observations on the increased attention manifested on the part of those responsible for the insane in providing them with employment and amusement, and in surrounding them with small comforts of domestic furniture.

The state of the asylum population in 1862 is shown in the accompanying table. On the first day of the year, the total number of pauper lunatics was 34,215; namely, in workhouses, 8803; in asylums and single patients, 25,412. The total number of private patients in asylums and hospitals at the same period was 5250, making the total *known* lunatic population at that date, 39,465.

---

